# Modeling users’ satisfaction and visit intention using AI-based chatbots

**DOI:** 10.1371/journal.pone.0286427

**Published:** 2023-09-08

**Authors:** Miguel Orden-Mejía, Mauricio Carvache-Franco, Assumpció Huertas, Orly Carvache-Franco, Wilmer Carvache-Franco

**Affiliations:** 1 Facultat de Turisme i Geografia, Universitat Rovira I Virgili, Vila-seca, Spain; 2 Universidad Espíritu Santo, Samborondón, Ecuador; 3 Department of Communication, Universitat Rovira I Virgili, Tarragona, Spain; 4 Facultad de Econonía y Empresa, Universidad Católica de Santiago de Guayaquil, Guayaquil, Ecuador; 5 Facultad de Ciencias Sociales y Humanísticas, Escuela Superior Politécnica del Litoral, ESPOL, Guayaquil, Ecuador; Shenzhen University, CHINA

## Abstract

AI-based chatbots are an emerging technology disrupting the tourism industry. Although chatbots have received increasing attention, there is little evidence of their impact on tourists’ decisions to visit a destination. This study evaluates the key attributes of chatbots and their effects on user satisfaction and visit intention. We use structural equation modeling with covariance procedures to test the proposed model and its hypotheses. The results showed that informativeness, empathy, and interactivity are critical attributes for satisfaction, which drive tourists’ intention to visit a destination.

## 1. Introduction

Technological evolution has transformed destination communication [1]. Smartphones and mobile device growth have changed how tourists seek and share information when traveling [2]. Different types of connectivity emergence, such as cloud computing and the Internet of Things [3], allow for the continuous and uninterrupted delivery of tourism information and services to users [4].

Smart Tourism Technologies (STT) have revolutionized the tourism industry [5]. Their rapid adoption generates substantial changes in communication with tourists, the travel experience, and traditional customer service. STT encompasses all technological applications that facilitate travelers’ experiences [6], adding value to destinations through connectivity, personalization, interaction, and co-creation [7,8]. Specific STT tools, products, and services range from smart devices, mobile applications, blockchain, sensors/actuators (beacons), smart cards, virtual reality (VR), augmented reality (AR), and chatbot or AI-based dialogue systems, among others [9].

This study focuses on chatbots, a term created from "chat" and "robot" [10]. Chatbots are software programs that simulate human conversation [11,12]. They are associated with productivity and profitability because they reduce the workload by being available 24/7.

In the travel context, global tourism brands and destinations use chatbots to communicate and improve the efficiency of tourism services management. As travelers spend more time on messaging platforms, chatbots will become the preferred interface for many activities that tourists typically perform through a specific website or application [13].

According to Forbes, 80% of companies currently use or will adopt chatbots to communicate with their users [14]. In the context of travel, the implementation of chatbots continues to increase steadily [15] due to the increase in digitized travel after the pandemic, the interconnected nature of the sector, the high flow of communication between providers and tourists, as well as the need to regularly manage user queries and requests [16]. As AI transforms business interactions with customers, more tourists receive their services through chatbot-based online or mobile channels. Therefore, research is needed to understand tourists’ experiences with chatbots.

On the other hand, research on chatbots has focused on the system’s design and architecture [17,18], the conceptual framework for adopting chatbots [16], the factors that predict the intention to use [19], and those that influence its continued use [20]. Interaction with chatbots not only improves satisfaction [21,22], but also purchases intention, and customer loyalty [23]. However, there is a lack of studies that analyze the crucial factors that predict a satisfactory experience when using a tourist chatbot during trip planning and how these factors influence the intention to visit the destination.

To achieve this, we designed a comprehensive model based on attribute theory [24,25]. The current study introduces the use of destination chatbots and explores how key chatbot factors (informativeness, empathy, accessibility, and interactivity) affect user satisfaction and visit intent. This research will improve the understanding of chatbots’ role in tourism, satisfaction backgrounds, and their effect on visit decisions. Additionally, it will guide DMOs, marketers, and chatbot designers in the development and adoption stages.

## 2. Literature review

Searching and sharing information through technologies can shape the tourist experience. Satisfactory tourist experiences positively influence a destination’s image [26] and the decision to visit a particular place [27]. Also, it is known that social media affects tourists’ visit decisions" [28–30]. Other researchers have confirmed the importance of STTs in generating a satisfactory tourist experience and increasing tourism and visiting decisions [31,32]. However, no studies have examined how destination chatbots can influence a tourist’s decision to visit a destination.

### 2.1 Theoretical foundation

Previous research has analyzed the attributes that measure the effectiveness of tourism technologies. For example, No and Kim [25] developed the theory of attributes and identified five constructs that evaluate the effectiveness of STTs: accessibility, trust, personalization, security, and interaction. Park and Gretzel [33] examined website quality on the willingness to use online travel agencies. The authors found six main attributes: compliance, information/content, ease of use, security/privacy, responsiveness, and visual appeal. Huang et al. [10] conducted a study recognizing the central role of STTs in travel planning to demonstrate that the attributes of smart tourism technologies are positively and significantly associated with exploration and exploitation, accessibility, and informativeness. Additionally, Jeong and Shin [6] conducted a study that evaluated STTs at a later stage of the trip. The researchers adopted four STT attributes found by [25] to examine their effects on traveler satisfaction and behavior intentions after the trip.

Furthermore, the STT attributes of informativeness, accessibility, interactivity, and personalization would be critical predictors for traveler satisfaction [34] and tourist destination loyalty [35]. Since the electronic service using chatbots is a recent advancement in the technological field, we used STT attributes as the theoretical basis for the current study; this allows for a more accurate understanding of destination chatbots, the factors that predict satisfaction, and the degree of visit intention after use.

### 2.2 Research model and hypothesis development

Just as empirical research is essential to understanding social media use patterns [36], we consider it imperative to analyze AI-based dialogue systems in tourism. Thus, this model suggests that the attributes of STT applied to chatbots represent the main predictors of user satisfaction. Additionally, the model tests whether gratification from experience with chatbots’ electronic services affects the intention to visit.

#### Informativeness

Informativeness is closely related to functionality [37,38], information quality [39], and performance expectancy [19]. Likewise, the quantity and quality of information provided by tourism technologies are fundamental factors in satisfying tourists’ needs and generating a good experience [40]. Accuracy, timeliness, concise nature, relevance, reliability, and completeness are other elements of informativeness [41,42]. Similarly, access to sufficient, truthful, accurate, up-to-date, and reliable information plays a decisive role [43] since the quality of the information stimulates user satisfaction [44]. In the context of chatbots, information quality can positively influence consumer satisfaction [45]. With this background, we believe that if the information provided by the chatbot is practical, relevant, and meaningful to the recipient, it will positively affect the overall satisfaction with the tool. Therefore, we propose the following hypothesis:"

H1: Informativeness in a DMO chatbot positively influence user satisfaction.

#### Empathy

In neuroscience, empathy is the ability to understand and respond to the emotional experiences of others [46]. Affective empathy is the ability to relieve another person’s emotional state, and cognitive empathy is the ability to know what another person is feeling [47]. Empathy is critical in tourism [48] since it generates better tourist experiences and avoids negative comments [49]. Moreover, empathic behavior is integral to the hospitality industry [50].

Tucker [50] compiled the existing literature on empathy and tourism. Studies about dark tourism [51,52] show that visits to places with suffering increase tourists’ empathy, just as historical empathy is enhanced by visiting historical places [53] or altruism through volunteer tourism [54].

Studies have concluded that technological agents who can show empathy and complex socio-emotional behavior generate more user confidence [55] and enhance the user experience [56]. Likewise, empathetic agents reduce user stress and stimulate greater engagement [57]. Similarly, chatbots that respond with empathetic tones significantly affect user satisfaction, reducing anxiety, frustration, and sadness [58].

However, despite all chatbots’ capabilities, they still do not meet users’ expectations [13,59]. Some research has focused on improving chatbot functionality and efficiency [33]. In contrast, others have contended that chatbots must include social abilities [59] and empathy [60], considering that technological agents must understand users, their motivations, feelings, and emotional states and act accordingly.

Studies about chatbots and empathy have increased recently [36,60,61] in order to make them more empathetic and human-like. Empathetic chatbots detect and understand the users’ emotions and respond to them on an appropriate emotional level [62]. However, more efforts should be devoted to understanding empathy and how chatbots can generate empathic responses instead of emotional responses [63]. That is why we pose the following hypothesis:

**H2:** Empathy in a DMO chatbot positively influence user satisfaction.

#### Accessibility

Accessibility means easy access to information technologies [27,42]. Ease of use predicts chatbots’ acceptance [45] and contributes to improving customer satisfaction [64]. Accessibility is associated with usability [65] and the generation of gratifying tourist experiences with technologies [9]. However, in a study by Jeong and Shin [6], accessibility was not a primary factor for tourists to enjoy a memorable experience from the destination.

Different studies have analyzed accessibility in tourism websites [66], blogs [67], or how tourists use innovative technologies in destinations [6]. Current research has focused on accessibility as one of the dimensions of STT [7,17,68], highlighting it as an essential factor for creating satisfactory tourism experiences. Therefore, we propose the following hypothesis:"

**H3**: Accessibility in a DMO chatbot positively influence user satisfaction.

#### Interactivity

Interactivity provokes favorable and positive attitudes in tourists [26]. Users perceive systems as interactive when reciprocal, responsive, and synchronous [69]. Interactivity is essential to increase the humanity of chatbot-based systems [70] and user engagement [8]. Furthermore, it is the most influential contributor to tourists’ memorable travel experiences [6]. Previous research on innovative end-user technologies has suggested that interactivity is an essential dimension of technology quality for smart services [71].

Interactivity is the level of continuous and immediate communication a tourist has during the trip using a smart technology system [27]. In chatbots, interactivity involves providing interactive conversations when assisting users. Additionally, interactivity can influence positive user reactions and increase chatbot service usage and satisfaction [27]. Therefore, we propose the following hypothesis:

**H4:** Interactivity in a DMO chatbot positively influence user satisfaction.

#### Relationships between satisfaction and visit intention

The friendly design of a website has a positive effect on the intention to visit the physical place [72]. In addition, it was shown that the attitude towards a destination website is associated with a positive user attitude towards that destination, which in turn will drive travel intention [73]. Even tourist satisfaction with STTs is essential to revisit the destination [74]. Previous studies have analyzed the satisfaction and experience generated due to smart technologies use and their influence on visit intention [6,9,75]. Therefore, we believe that the tourists’ positive cognition of the destination as a background, added to a satisfactory experience with the chatbot, could increase the interest in revisiting the destination. Thus, we pose the following hypothesis:

H5: User satisfaction with a chatbot is positively associated with the tourists’ visit intentions.

**Fig 1:**
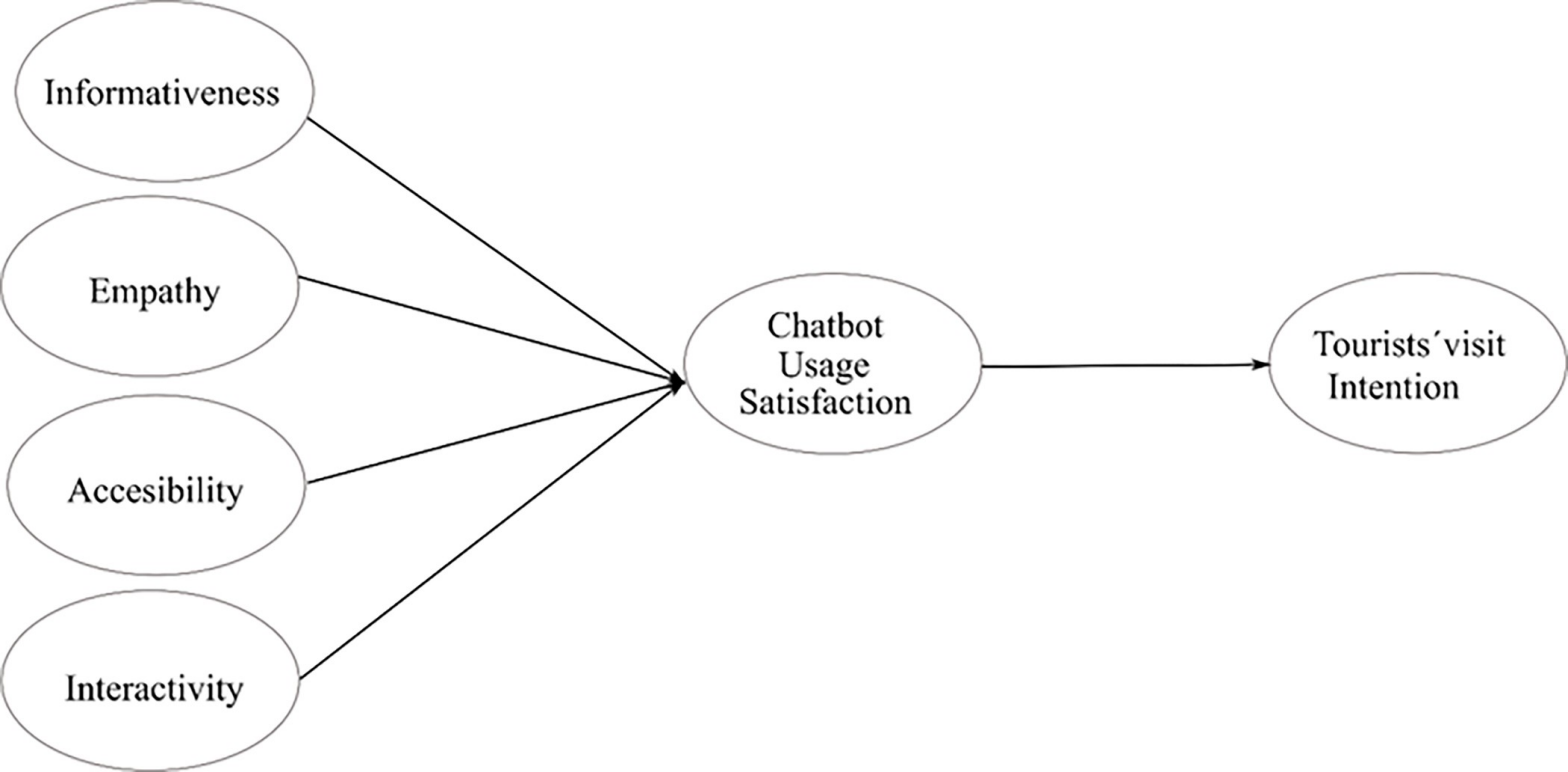
Proposed model.

Fig 1 shows the proposed model.

## 3. Methodology

### 3.1. Case studies

For this study, we selected the chatbots "TurismodeMurcia" and "Victoria la Malagueña," two of the main Spanish DMOs providing tourist information about destinations through chatbots. The latter even received The Chatbot Tourism Awards 2019 from the State Mercantile Society for the Management of Tourism Innovation and Technologies (SEGITTUR). The primary function of these chatbots is to provide information about leisure activities, tourist attractions, and tourist infrastructure. The "Victoria la Malagueña" chatbot can be accessed through Facebook Messenger or Google Assistant, and the "Turismo de Murcia" chatbot can be via Telegram.

### 3.2. Data collection

Young students from Rovira Virgili University, Spain, were the sample for this study. We conducted an experiment that was divided into three phases. First, we requested permission to enter the university’s different classrooms to socialize the experiment. Once in the classroom, we presented the study without revealing its objectives to avoid biases and explained the characteristics of tourism technologies, including chatbots. Second, the students were asked to interact for 10 minutes with the chatbots from the cities of Malaga and Murcia, imagining that they were visiting them on their next vacation. During the human-chatbot conversational session, students could request information about the destination regarding restaurants, attractions, or tourist services. Third, after interacting with the chatbot, participants completed a questionnaire about their experience with the chatbot.

The data were collected between October and November 2019. The sampling approach was non-probabilistic, convenience sampling. This method was chosen because it is frequently used in tourism and chatbots [76,77]. However, it is essential to mention that inferences can only be made about the group of participants, not the population at large, so the results may not be generalizable. Finally, after verifying outliers and missing data and excluding invalid questionnaires, we used 469 valid questionnaires out of 483 respondents for the final analysis.

### 3.3. Survey design

The first part of the survey contained questions about the respondents’ sociodemographic characteristics. The second block had items measuring the constructs of the study. Our survey items were either adapted from the literature or developed based on the conceptual definitions of extant studies. For example, we adapted the items from previous research on informativeness, accessibility, and interactivity [26,27,78,79], empathy [34,59], and tourists’ visit intention [80]. All items had a multi-item measure, anchored on a scale ranging from 1 = strongly disagree to 7 = strongly agree: six items for informativeness, three for accessibility, six for empathy, and four for intention to visit. We also measured chatbot usage satisfaction based on Lin and Hsieh [81], with four items for this construct. We used a 7-point Likert scale: one was ’not at all satisfied’ and seven was ’very satisfied.’ Table 1 provides the operational definitions of the variables used in our research model based on the aforementioned studies. We validated the questionnaire by conducting a pilot test with 25 respondents to facilitate the readability and understanding of the items. The questionnaire presented minimal corrections.

**Table 1:** Operational definitions.

Construct	Operational definition	Reference
Informativeness	The degree to which a tourist receives useful, relevant, or updated information while using a chatbot.	Pavlou et al. [79]; Kim and Niehm [78],
Empathy	The degree to which users perceive that chatbots can engage in empathetic conversations with humans.	Zhou et al. [34]; Chaves & Gerosa [59]
Accessibility	The degree to which a tourist accesses the information without complication while using a chatbot.	No and Kim [27]
Interactivity	The degree to which a user perceives that their communication with a chatbot resembles their dialogues with human agents and the ease of sharing content.	No and Kim [27]
Satisfaction	The degree to which a tourist is satisfied with chatbot use.	Lin and Hsieh [81]
Intention to visit	The degree to which a tourist intends to visit a destination after using the chatbot.	Assaker and Hallak [80]

Since common method variance [CMV] [82] is often affected by the method’s complexity, item placement, and scale, we systematically examined the construction of the items to avoid ambiguous, vague, and unknown terms [83]. Additionally, we separated the predictor variables measures and the response variables [84].

We applied Harman’s one-factor test by entering all the elements of the constructs in an exploratory factor analysis. The result was a multifactorial solution, where the first factor explained 41.37% of the total variance, below the threshold of 50% [84]. Therefore, the data had no significant common method bias, and they were ready for further analysis.

### 3.4. Data analysis

A descriptive analysis summarized the characteristics of the respondents’ sociodemographic profiles. The principal analysis to test the hypotheses was a two-step structural equation modeling (SEM) using SPSS version 25 and AMOS version 24. First, we carried out confirmatory factor analysis (CFA) to assess the quality of the measurement. Second, using a maximum likelihood estimator, we applied SEM to test the hypothesized relationships between the latent variables [85]. Additionally, we used various fit indices such as the root mean square error of approximation (RMSEA), the comparative fit index (CFI), the normed fit index (NFI), the incremental fit index (IFI), and Tucker-Lewis index (TLI).

## 4. Empirical results

### 4.1. Sample description

The participants had an age range between 18 and 24 years old (94.5%); 70.6% were women (n = 331), and 29.2% were men; 68.6% of them dedicated between 3 to 5 hours to internet entertainment; and 51.8% travel for tourism once a year, or twice a year 22.6%. Furthermore, topics related to gastronomy (restaurants, coffee shops) were the most interesting for the participants, followed by the gastronomic routes, opening and closing times of museums and theaters, and the public transport system schedules. Table 2 shows the results.

**Table 2:** Profile of survey respondents.

Categories	Frequency	%
Gender		
Male	137	29.2
Female	331	70.6
Age, years		
18–24	443	94.5
25–30	19	4.1
31–35	3	0.6
> 35	4	0.9
Internet		
1 hour	14	3.0
2 hours	80	17.1
3 hours	191	40.7
5 hours	131	27.9
6–8 hours	53	11.3
Tourism		
Every 3 years	35	7.5
Every 2 years	37	7.9
Once a year	243	51.8
Twice a year	106	22.6
Three times a year	48	10.2
Chatbot queries		
Gastronomy	239	51
Tourist routes	61	13
Museums	38	8.1
Transportation Schedule	37	7.9
City monuments	28	6
City theaters	14	3
Others	52	11

### 4.2 Structural model evaluation

The measurement model had an acceptable fit, according to CFA (*λ*^2^ = 912.182; degrees of freedom [*df*] = 362; *λ*^2^/*df* = 2.520) at a level (*p* = 0.001). Also, the fit indices showed a good model fit to the data (CFI = .950; TLI = .944; NFI = .921; IFI = .951; RMSEA = .057) with CFI, NFI, and IFI values greater than 0.90 and an RMSEA value smaller than 0.08 [86]. As seen in Table 3, the individual reliability analysis of the indicators shows that most have factor loadings (λ) greater than 0.7, which is the acceptable threshold [87]. Although, Barclay et al. [88] argue that loads greater than 0.50 or 0.60 may be acceptable when validated scales have been used and applied in previous studies and different fields. In addition, all latent variables reached convergent validity because the AVE means exceeded the 0.5 threshold, except in the interactivity construct, showing that it shared more variance with its indicators than error variance [89]. However, Malhotra and Dash [90] point out that AVE is often too strict and that reliability can be established only through composite reliability.

**Table 3:** Assessment of the measurement model.

Construct and associated ítems	Mean	SD	Factor Loading
**INF: Informativeness** (CR = 0.93; α = 0.935; AVE = 0.70)			
INF1: Provided adequate information	3.54	1.64	.817***
INF2: Provided high-quality information	3.47	1.62	.857***
INF3: Provided detailed information	3.77	1.52	.811***
INF4: Provided useful information	3.87	1.71	.887***
INF5: Provided substantial information	3.88	1.68	.848***
INF6: Provided relevant information	3.68	1.73	.817***
**EMP: Empathy** (CR = 0.908; α = 0.905; AVE = 0.623)			
EMP1: It was fun when it answered.	3.44	2.09	.778***
EMP2: I was impressed when it answered.	3.42	1.95	.848***
EMP3: I smiled at the answers.	3.93	2.13	.816***
EMP4: I liked chatting with the chatbot.	3.56	1.85	.841***
EMP5: The chatbot used emotional expressions.	3.90	2.02	.634***
EMP6: Overall, Victoria showed empathy when chatting (in the chat-based encounter).	3.41	1.83	.798***
**AC: Accessibility** (CR = 0.883; α = 889; AVE = 0.679)			
AC1: I can log in at any time.	4.84	1.65	.846***
AC2: It has an easy-to-use interface.	4.65	1.71	.906***
AC3: I can access it without complicated registrations.	4.38	1.76	.848***
AC4: I can log in anywhere.	4.46	1.83	.680***
**INT: Interactivity** (CR = 0.743; α = 0.742; AVE = 0.491)			
INT2: The chatbot responds to my requests promptly.	4.22	1.48	.705***
INT3: The chatbot always answered my questions.	4.43	1.27	.708***
INT4: It is easy to share information with the chatbot.	4.31	1.35	.690***
**SAT: Satisfaction** (CR = 0.939; α = 0.938; AVE = 0.723)			
SAT1: I am satisfied with the use of the chatbot.	4.36	2.06	.903***
SAT2: I am pleased with the experience of chatbots.	4.27	2,12	.929***
SAT3: The chatbot exceeded my expectations.	4.37	1.97	.877***
SAT4: The chatbot is close to my ideal tourism technology.	4.44	2.04	.858***
SAT5: I think using the chatbot on my trip is a good idea.	4.05	1.92	.855***
SAT6: Overall, I am satisfied with the chatbot application.	3.44	1.66	.650***
**IN: Visit intention** (CR = 0.941; α = 0.940; AVE = 0.801)			
(after chatting with the chatbot)			
IN1: I intend to visit the destination after experiencing the chatbot service.	2.75	1.72	.863***
IN2: I will visit the destination after experiencing the chatbot service.	2.63	1.61	.923***
IN3: I will likely visit the destination in the future after experiencing the chatbot application.	2.80	1.71	.928***
IN4: I want to recommend the destination to others after experiencing the chatbot application.	2.79	1.70	.864***

M = Mean

SD = Standard Deviation

∝ = Cronbach’s alpha

CR = Composite Reliability

AVE = Average Variance Extracted

*** Significant at 1%.

This study tested discriminant validity because all intra-construct correlations were lower than the square root of each construct’s AVE. In other words, all indicators were better explained by their respective construct than by the other constructs (see Table 4). Furthermore, Cronbach’s Alpha and Composite Reliability values confirm the internal consistency for each construct, higher than the minimum level of 0.7 [89]. The HTMT values are shown in Table 5.

**Table 4:** Result of discriminant validity (Fornell-Larcker).

Construct	INF	EMP	AC	INT	SAT	IN
INF	**0.840**					
EMP	0.670***	**0.789**				
AC	0.230***	0.065	**0.824**			
INT	0.638***	O.688***	0.091	**0.701**		
SAT	0.594***	0.677***	-.257***	0.584***	**0.850**	
IN	0.619***	0.591***	0.106*	0.413***	0.457***	**0.895**

Note: The square root of AVEs is shown diagonally in bold.

**Table 5:** Result of discriminant validity (HTMT).

Constructs	INF	EMP	AC	INT	SAT	IN
INF						
EMP	.674					
AC	.246	.075				
INT	.634	.692	.113			
SAT	.627	.702	.246	.590		
IN	.623	.596	.115	.420	.501	

**Fig 2:**
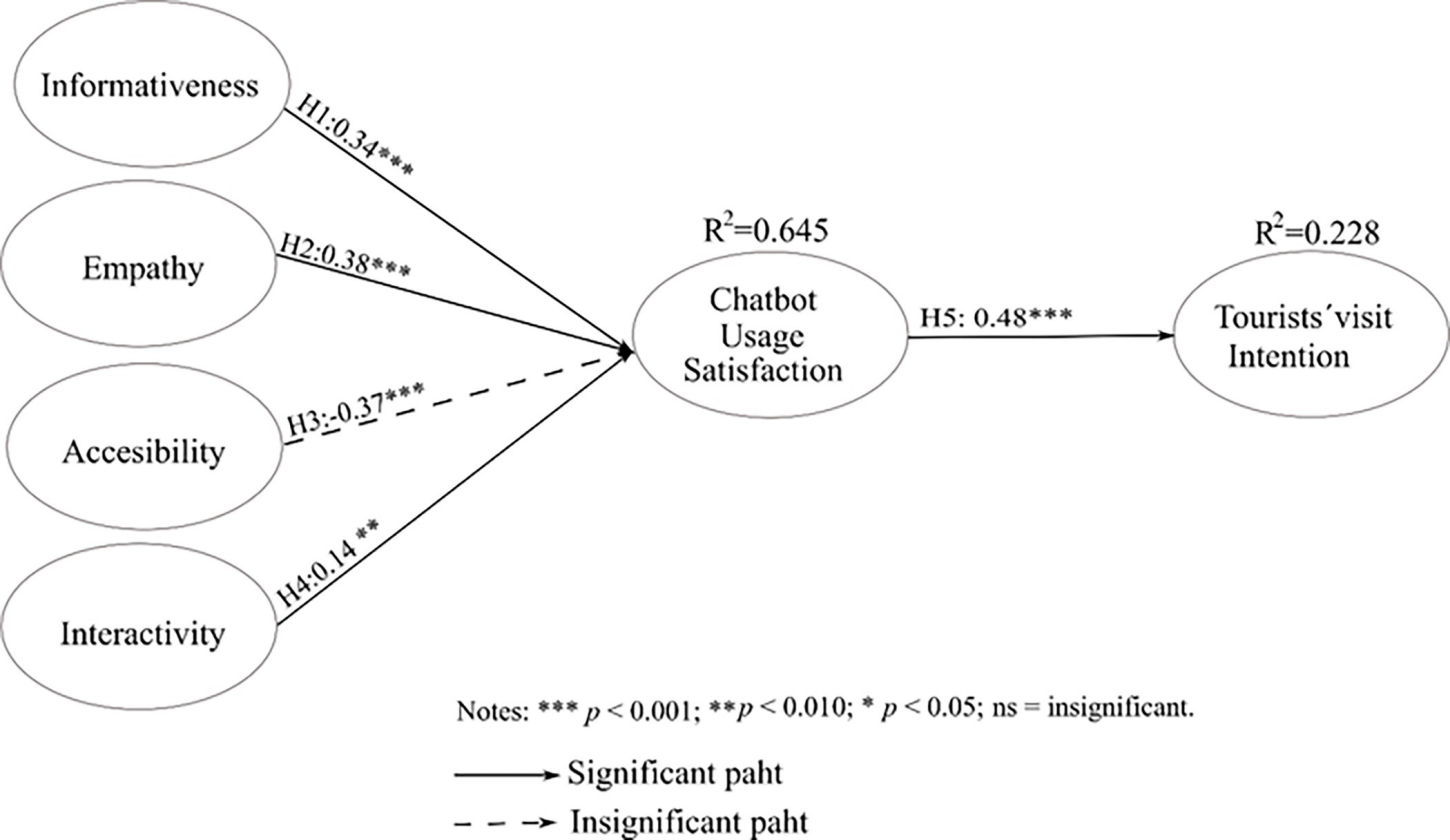
Result of the structural model estimation.

Fig 2 shows the relationships between constructs. Perceived empathy (EMP) had a positive influence on users’ satisfaction (β = 0.38; *p* <0.001), supporting H2. Empathy is the analyzed chatbot attribute that most influenced satisfaction. This result shows that empathetic chatbots help create a more attractive image of the destination and generate greater user satisfaction.

The significant analysis of the standardized regression coefficients revealed that informativeness (INF) in the interaction with a chatbot positively impacted and had a high effect on user satisfaction (β = 0.34; p < 0.001), which supports H1. In other words, the more appropriate the information a DMO chatbot offers, the greater the satisfaction. On the other hand, the proposed positive relationship between accessibility (AC) and user satisfaction with a chatbot was not significant, leading to a rejection of hypothesis H3 (β = -.37; *p* <0.010). This unexpected finding shows that users are not influenced by the accessibility of a chatbot when trying to get to know a destination. Furthermore, the relationship between interactivity (INT) and user satisfaction with the chatbot was significant (β = 0.14; *p* <0.01), which supports H4.

Finally, user satisfaction with the chatbot positively affected tourists’ visit intention (β = 0.48; *p* <0.01); thus, H5 is supported. The coefficient of determination of the user satisfaction construct was *R*^2^ = 0.645, with a good predictive rate, and tourists’ visit intention was *R*^2^ = 0.228 (See Table 6).

**Table 6:** Result of hypothesis testing.

Hypotheses		Paht Coefficient	Sig.	Standard Error	*t* Value	Supported
H1	INF → SAT	.34	***	0.098	6.551	**Supported**
H2	EMP → SAT	.38	***	0.106	6.734	**Supported**
H3	AC → SAT	-.37	***	0.067	-10.14	*Unsupported*
H4	INT → SAT	.14	**	0.112	2.320	**Supported**
H5	SAT → IN	.48	***	0.037	10.364	**Supported**

n = 469

Sig: Significant

** p < .01

***p < .001.

## 5. Discussion

### 5.1. Research findings

This research demonstrates which chatbot attributes have the most influence on user satisfaction and their effect on the intention to visit a destination. The results suggest several essential aspects to consider when designing chatbots that assist tourists during trip planning.

Firstly, the structural model suggests that informativeness, empathy, and interactivity attributes significantly influence user satisfaction. In this study, informativeness is a critical predictor in generating satisfactory tourist experiences [40], consistent with previous literature [41,43,45]. In tourism and conversational technologies, informativeness is essential in the design and implementation of chatbots, as these systems are designed to provide information and respond to questions or requests accurately and efficiently. Tourists expect chatbots to provide updated, relevant, detailed, and valuable information to address their doubts or problems before, during, and after the trip.

"Second, an important contribution of this research is the empathy attribute, configured as an additional construct to those found in previous studies related to STT [10,26,27]. This study’s SEM model suggests that empathy best explains user satisfaction, corroborating previous research [58,91]. Chatbots with empathetic characteristics significantly improve interaction with tourists, as they can help create more personalized, enjoyable, and efficient experiences for the user. Responding empathetically to needs and concerns will make tourists feel more comfortable and welcome, increasing their satisfaction. Therefore, chatbots should acquire social skills and increase their ability to detect and understand users’ emotional states through conversational tone and language analysis to respond accurately and empathetically, thus leading to trust [34,55], reducing stress [57] and improving user experience [56].

The hypothetical relationship between accessibility and user satisfaction is not confirmed. On the one hand, these results contradict previous studies that showed that accessibility in ICT is an essential factor in creating satisfying tourist experiences [7]. On the other hand, this finding aligns with those reported by Jeong and Shin [6], who detected that accessibility in STTs has an insignificant impact on memorable tourist experiences with the destination due to the high current technological infrastructure of smart tourist destinations, equipped with high-capacity bandwidth. In addition, the results of this study are consistent with previous research [24,92] that measured the ease of using chatbots through the effort expectancy factor, similar to the accessibility factor.

Interactivity in chatbots, an advanced feature of intelligent services, is essential to provide tourists with a good experience. In this research, interactivity positively impacts user satisfaction, consistent with preliminary studies [25] that determined that interactivity can influence users’ positive reactions and eventually increase their satisfaction and continued use of chatbot services. However, in our study, finding marginal support in the relationship between interactivity and satisfaction indicates that a more rigorous attempt is needed to confirm this relationship in future studies empirically.

Chatbots must be designed to interact effectively with users and be able to provide quick responses to tourists’ questions so that they do not feel frustrated or bored waiting for responses. The chatbot response speed is a perceived interactive element [69] that elicits positive tourist attitudes [26] and increases chatbots’ humanity’s perception [70]. However, in recent studies in the context of Chinese online travel agencies, the responsiveness and chatbot response speed may not be a priority from the perspective of Chinese users [25] because human services in China are relatively fast compared to other countries.

Furthermore, the chatbot interface should be intuitive so that the information they obtain during the message exchange is easy to share. Therefore, destination chatbots that aim to enhance the tourist experience through a dialogue system must constantly improve interactivity.

As tourists have a satisfactory experience with a destination chatbot, they are likelier to feel that the information and attention received are personalized and tailored to their needs which can increase their confidence in the destination and their desire to visit it. Additionally, it may influence the overall perception of the destination. The results of this study have confirmed that user satisfaction is crucial in the intention to visit the destination. Such findings are consistent with previous research demonstrating how satisfaction with the experience of technology directly influences the intention to visit [9,73,75].

## 6. Practical implications

The first practical implication of this research is related to the quality of chatbot responses. This study recommends that designers further improve the informativeness attribute by advancing toward computational modeling to refine message understanding to generate the most accurate responses possible. Therefore, AI-based chatbot designers should venture into an "open domain" using transformer models (architecture that allows predicting text from the relationship of previous words). Additionally, algorithm training could be based on tourism platforms with conversation samples so that the chatbot can extract information and learn to map conversation parameters and patterns in travel contexts. With this strategy, the chatbot’s neural network architecture will be able to learn better and respond more accurately to tourist requirements.

Another implication for chatbot designers is related to functionality and empathetic responses. An empathetic chatbot should link cognitive and behavioral abilities, similar to the computational model of empathy for interactive agents [93]. Incorporating artificial intelligence with the ability to detect user emotions and respond empathetically could positively impact tourists’ perception of the destination, which could lead to an improvement in the destination image and intention to visit.

The study suggests that chatbot designers should train algorithms in contingent responses. In a human-chatbot conversation, chatbot responses should be subject to initial and subsequent messages. This feature would make users more interactive with the chatbot, as it mimics the conversations underlying contingency between people. Additionally, a good conversation flow will help chat agents be perceived as more human, producing greater satisfaction with their use.

## 7. Theoretical implications

The theoretical implications of this study are twofold. First, an integrated model was developed between chatbot attributes, user satisfaction, and behavioral intentions related to the likelihood of visiting a destination after using chatbots. Significant relationships were found between interactivity, informativeness, and empathy with satisfaction, impacting tourists’ intentions to visit a destination. This study adds to existing theory by showing that user satisfaction is a precursor to the intention to visit a destination when using a chatbot.

Secondly, one of the main contributions of this study to the academic literature on tourism and technologies is the development of the empathy attribute. Empathic abilities are already present in STT and dialogue systems like chatbots but have not been thoroughly investigated. This research focused on constructing and validating an underlying structure that explains the empathic ability of chatbots to fill this gap. The development of the empathy factor is a generic contribution to the attributes that measure the effectiveness of STT and suggests an extension to preliminary studies. Thus, STT, in general, and chatbots, in particular, have a new attribute that will serve as a measurement tool for future research related to tourism and conversational technologies. Therefore, empathy becomes another element to consider when analyzing destination chatbots and intelligent tourism technologies, demonstrating its significant influence on tourist decisions.

## 8. Conclusions

The study shows that chatbot technology influences tourists’ visit decisions, and user satisfaction is a critical predictor for destination choice. We interpret the causal relationships between chatbot attributes, satisfaction, and their effect on tourists’ visit intention through a structural model.

Empathy, informativeness, and interactivity are the main attributes influencing satisfaction. It was found that empathy significantly impacts user satisfaction, which in turn leads to tourists’ intention to visit the destination. That is, having a satisfying chatbot experience based on emotional dialogues with empathetic tones is a powerful attribute that often influences a tourist’s decision to choose a destination. Empathy is more influential, as it was found that users value a friendly and empathetic conversation much more than the quality of responses (informativeness) or ease of content sharing (interactivity). In conclusion, empathetic message exchanges help create satisfying experiences that impact tourists’ decisions to visit smart destinations such as Malaga and Murcia.

As mentioned earlier, empathy is crucial in generating satisfying tourist experiences and is a key predictor for humanizing conversations with chatbots. It is important to note that a factor related to empathetic ability had not yet been contemplated in the theory of STT attributes. Therefore, this study serves as the first contribution to thoroughly analyze the effect of empathetic responses from chatbots on user experience and visit intention.

Finally, there are still critical challenges to promote empathy in technology-oriented tourism as advances in social computing, affective computing, natural language processing, and machine and deep learning techniques are still in their early stages. However, the likelihood of achieving satisfactory human-machine interaction by building a human cognitive-neuronal system in a machine is closer.

## 9. Limitations and future research

This study needs to acknowledge some limitations. Firstly, the participant sample was limited to university students, mostly young people under 24. This sample of potential young tourists was chosen as a critical segment for this type of study. However, replicating it with participants of all ages could yield different results.

The fact that the experiment and survey were not conducted at the actual destination also posed a limitation. Therefore, conducting an exploratory study with tourists who have used the chatbot before and during their visit to Málaga or Murcia would be interesting.

The choice of non-probabilistic sampling is a limitation in generalizing the results; thus, it is suggested to conduct a study with probabilistic sampling with age and gender heterogeneous samples. A sample of tech-savvy participants and their counterparts could also be used to understand the technology acceptance of travelers and how it influences satisfaction and intention to continue using the tool.

Finally, it should be noted that the machine learning algorithms of the studied chatbots were in their initial stages, and therefore their comprehension abilities were still limited. However, over time and with training, they could improve the quality of the information provided and how they respond to users.

This study could be replicated in smart destinations in developing countries with technological asymmetries to contrast these results allowing for even more interesting and insightful contributions to the current literature. Furthermore, future research could focus on constructing a scale to measure the empathy of chatbots from a cognitive and affective perspective to explore new areas of knowledge in communication, psychology, and tourism.

## Supporting information

S1 File (SAV)
